# Deacetylation and Desuccinylation of the Fucose-Rich Polysaccharide Fucopol: Impact on Biopolymer Physical and Chemical Properties

**DOI:** 10.3390/molecules27217165

**Published:** 2022-10-23

**Authors:** Sílvia Baptista, Diana Araújo, Patrícia Concórdio-Reis, Ana C. Marques, Elvira Fortunato, Vítor D. Alves, Filomena Freitas

**Affiliations:** 1 Associate Laboratory i4HB-Institute for Health and Bioeconomy, School of Science and Technology, NOVA University Lisbon, 2829-516 Almada, Portugal; 2 UCIBIO—Applied Molecular Biosciences Unit, Department of Chemistry, School of Science and Technology, NOVA University Lisbon, 2829-516 Almada, Portugal; 3 73100, Lda. Edifício Arcis, Rua Ivone Silva, 6, 4° piso, 1050-124 Lisboa, Portugal; 4 i3N/CENIMAT, Department of Materials Science, Faculty of Sciences and Technology, NOVA University of Lisbon and CEMOP/UNINOVA, 2829-516 Almada, Portugal; 5 LEAF—Linking Landscape, Environment, Agriculture and Food, Associated Laboratory TERRA, Instituto Superior de Agronomia, Universidade de Lisboa, Tapada da Ajuda, 1349-017 Lisboa, Portugal

**Keywords:** deacetylation, desuccinylation, alkaline treatment, polysaccharide, FucoPol, rheology, emulsion, film forming

## Abstract

FucoPol is an acylated polysaccharide with demonstrated valuable functional properties that include a shear thinning fluid behaviour, a film-forming capacity, and an emulsion forming and stabilizing capacity. In this study, the different conditions (concentration, temperature, and time) for alkaline treatment were investigated to deacylate FucoPol. Complete deacetylation and desuccinylation was achieved with 0.02 M NaOH, at 60 °C for 15 min, with no significant impact on the biopolymer’s sugar composition, pyruvate content, and molecular mass distribution. FucoPol depyruvylation by acid hydrolysis was attempted, but it resulted in a very low polymer recovery. The effect of the ionic strength, pH, and temperature on the deacetylated/desuccinylated polysaccharide, d-FucoPol, was evaluated, as well as its emulsion and film-forming capacity. d-FucoPol aqueous solutions maintained the shear thinning behaviour characteristic of FucoPol, but the apparent viscosity decreased significantly. Moreover, contrary to FucoPol, whose solutions were not affected by the media’s ionic strength, the d-FucoPol solutions had a significantly higher apparent viscosity for a higher ionic strength. On the other hand, the d-FucoPol solutions were not affected by the pH in the range of 3.6–11.5, while FucoPol had a decreased viscosity for acidic pH values and for a pH above 10.5. Although d-FucoPol displayed an emulsification activity for olive oil similar to that of FucoPol (98 ± 0%) for an oil-to-water ratio of 2:3, the emulsions were less viscous. The d-FucoPol films were flexible, with a higher Young′s modulus (798 ± 152 MPa), a stress at the break (22.5 ± 2.5 MPa), and an elongation at the break (9.3 ± 0.7%) than FucoPol (458 ± 32 MPa, 15.5 ± 0.3 MPa and 8.1 ± 1.0%, respectively). Given these findings, d-FucoPol arises as a promising novel biopolymer, with distinctive properties that may render it useful for utilization as a suspending or emulsifier agent, and as a barrier in coatings and packaging films.

## 1. Introduction

FucoPol is a water-soluble anionic heteropolysaccharide secreted by the Gram-negative bacterium *Enterobacter* A47 (DSM 23139) composed of fucose, galactose, glucose, and glucuronic acid (2.0:1.9:0.9:0.5 molar ratio) with a high molecular weight (1.7–5.8 × 10^6^ Da) and an intrinsic viscosity value of 8.9 dL/g [1,2]. Structurally, it comprises a main chain composed of a →4)-α-L-Fucp-(1→4)-α-L-Fucp-(1→3)-β-D-Glcp(1→ trimer repeating unit, and a trimer branch α-D-4,6-pyruvyl-Galp-(1→4)-β-D-GlcAp-(1→3)-α-D-Galp(1→ in the C-3 of the first fucose [3]. It is an acylated polysaccharide, containing 13–14 wt% pyruvyl, 3–5 wt% acetyl, and 2–3 wt% succinyl as substituent groups [2]. Glucuronic acid, pyruvate, and succinate confer FucoPol an anionic nature that promotes its interaction with charged molecules [2]. FucoPol’s aqueous solutions present a shear-thinning behaviour, with viscoelastic properties comparable to those of guar gum and fucogel [1]. Furthermore, FucoPol possesses valuable functional properties that include an emulsion forming and stabilizing capacity [1,2,4], a film forming [4,5] and gelling capacity [6], as well as biological properties, such as a wound healing ability [7], photoprotection [8], an antioxidant capacity [9], and cryoprotection [3], that potentiate its utilization in several applications.

Interchain and/or intrachain interactions between polysaccharide molecules are greatly affected by their degree of substitution and the type of substituent groups [10,11]. The presence of organic acyl groups, such as acetyl, pyruvyl, succinyl, among others, are important for the polysaccharides’ functional properties, including their viscoelastic properties, gelling ability, and bioactivity [10]. Well-studied deacylated polysaccharides include gellan and xanthan gums. For gellan gum, deacetylation induces the formation of harder gels with a higher thermal stability [12] than an acetylated biopolymer. It also improves the capacity to form hydrogels when mixed with cationic surfactants [10], and promotes cation-induced gelation in the presence of Ca^2+^ in a physiological environment [13]. For xanthan gum, deacetylation increases the gel strength and viscoelasticity [14,15], as well as the formation of films with a higher water absorption behaviour when compared to the acetylated form [16]. Ridout et al. [17] studied the removal of succinyl and acetyl in succinoglycan polysaccharide. Succinyl removal improved the shear thinning behaviour of the aqueous solutions, while increasing the biopolymer’s order-disorder transition cooperativity. When acetyl removal was performed, the order-disorder transition temperature decreased, whereas the succinyl removal increased this physical property. 

Deacetylation and desuccinylation can be achieved using chemical (e.g., alkaline treatments at a high temperature and/or a high pressure [18]), enzymatic [19], or physical (e.g., ultrasound) treatments [20]. Guetta et al. [21] obtained deacetylated Fucogel by alkaline conditions using 0.1 M NaOH for 30 min at 80 °C. For chitin deacetylation, Kjartansson et al. [20] used 12.5 M NaOH treatment for 4 h at 100 °C. Pinto et al. [22] used NaOH and KOH 0.01 M at 25 °C for 3 h to obtain deacetylated xanthan gum.

This study focused on the development of a procedure for the removal of acetyl, succinyl, and pyruvyl substituent groups from the FucoPol macromolecule. The removal of these substituent groups from FucoPol was not studied so far. Alkaline treatments with NaOH were designed and tested for FucoPol’s deacetylation/desuccinylation, while an acid treatment with hydrochloric and oxalic acids was attempted for the pyruvate removal. The impact of the applied treatments on the polysaccharides’ physical-chemical properties were evaluated, including the rheological properties, emulsifying behaviour, and film-forming capacity. 

## 2. Materials and Methods

### 2.1. FucoPol Production and Extraction

FucoPol was produced by the bioreactor cultivation of *Enterobacter* A47 (DSM 23139), as described by Torres et al. [23], and extracted from the cell-free supernatant by the diafiltration/ultrafiltration procedure described by Baptista et al. [2]. 

### 2.2. Chemical Deacylation of FucoPol

FucoPol deacetylation and desuccinylation studies under alkaline conditions were based on the procedures described by Pinto et al. [22] and Lima et al. [24], by subjecting the polysaccharide to different NaOH concentrations (0.005, 0.01, 0.02, and 0.05 M), temperatures (40 and 60 °C), and reaction times (15, 30 and 60 min). Briefly, the FucoPol samples (5 mg) were dissolved in deionized water (5 mL) and contacted with NaOH, under a controlled temperature. After the treatment, the samples were cooled to room temperature and extensively dialysed using a 12 kDa MWCO membrane (ZelluTrans/Roth) against deionized water to eliminate the low molecular weight compounds resulting from the hydrolysis procedure and salts. Finally, the samples were freeze-dried (ScanVac CoolSafeTM, LaboGene, Lillerød, Denmark), at −110 °C for 48 h. 

Depyruvylation studies were undertaken on the deacetylated/desuccinylated FucoPol by its hydrolysis with hydrochloric or oxalic acids, following the procedures described by Pinto et al. [22] and Herasimenka et al. [25], respectively. Hydrolysis was achieved using FucoPol solution (1.0 wt%) in hydrochloric acid (2.0 M) at 80 °C for 16 h. The hydrolysis with oxalic acid (0.5 M) was performed using FucoPol solution (0.1 wt%) at 100 °C for 2 h. The samples were dialysed and freeze-dried as described above.

All assays were performed in duplicate.

### 2.3. Physical and Chemical Characterization 

#### 2.3.1. Composition

The monosaccharides profiles of the deacetylated/desuccinylated FucoPol (herein named d-FucoPol), as well as the original FucoPol were determined by liquid chromatography, using a Carbopac PA10 column (Thermo Scientific™ Dionex™, Sunnyvale, CA, USA), equipped with an amperometric detector. The analysis was performed at 30 °C with NaOH 4 mM as the eluent, at a flow rate of 0.9 mL/min. Fucose, glucose, galactose, and glucuronic acid, at a concentration between 5 and 100 ppm, were used as the standards. The acyl substituents groups, namely acetate, succinate, and pyruvate, were quantified by liquid chromatography, using an Aminex HPX-87H 300 × 7.8 mm column (Biorad, Hercules, CA, USA) connected to an infrared (IR) detector, using sulfuric acid 0.01 N as the eluent, at a flow rate of 0.6 mL/min, at 30 °C. Acetic, succinic, and pyruvic acid were used as the standard at a concentration between 25 and 500 ppm. 

#### 2.3.2. Fourier Transform Infrared analysis 

Fourier Transform Infrared (FT-IR) spectroscopy with Diamond ATR (attenuated total reflectance) was applied to the spectra collection of the samples with a Perkin Elmer Spectrum Two (Perkin Elmer Inc., Waltham, MA, USA), equipped with a lithium tantalate detector with an SNR (signal to noise ratio) of 14.500:1. The resolution was 0.5 cm^−1^ with eight scans. The samples were positioned in the absorbance chamber and corrected by applying the ATR-correction function of Perkin Elmer Spectrum (Waltham, MA, USA) software in the region of 4500–500 cm^−1^ [2].

#### 2.3.3. Molecular Mass Distribution 

The average molecular weight (Mw), molecular number (Mn), and polydispersity index (PDI = Mw/Mn) were determined by size exclusion chromatography (SEC). The samples were dissolved in deionized water (2–4 mg/mL) and injected in a size exclusion-high-performance liquid chromatography (SE-HPLC) system (Knauer, Smartline system 1000, Berlin, Germany), equipped with a Phenomenex Phenogel Linear LC Column (300 mm × 7.8 mm, Knauer) coupled to a refractive index detector (RI detector, Knauer Smartline 2300). The analysis was performed at 25 °C with LiNO_3_ 0.1 M as the eluent, at a flow rate of 0.6 mL/min and an injection volume of 50 µL. The Mw was estimated after a universal calibration with the pullulans (≤600 kDa).

#### 2.3.4. Thermal Properties

Thermogravimetric analysis (TGA) was performed using a Thermogravimetric Analyzer Labsys EVO (Setaram, Lyon, France). The samples were arranged in aluminium crucibles and heated until 550 °C, with a heating rate of 10 °C/min, in air. The thermal degradation temperature (T_deg_, °C) corresponds to the temperature value obtained for the maximum decreasing peak of the sample mass [2].

#### 2.3.5. Intrinsic Viscosity

The intrinsic viscosity (*η*) was determined by double extrapolation to zero concentration of the Huggins and Kraemer equations, as described by Freitas et al. [1]. The capillary viscosity measurements were performed on an automatic viscosity measuring unit AVS 450 (Schott-Gerate, Mainz, Germany), with an Ubbelhode capillary viscometer (Ref. 53013/Ic, Schott-Gerate, Mainz, Germany) immersed in a water bath at a constant temperature (25 ± 0.5 °C). 

### 2.4. Apparent Viscosity and Viscoelastic Properties

Aqueous d-FucoPol solutions (0.75, 1.5, and 3.0 wt%) were prepared in 0.1 M NaCl. For the 3 wt% d-FucoPol solution, different pH values (3.6, 4.8, 6.2, 8.5, and 11.5), temperatures (10, 25, 45, 65, and 80 °C), and ionic strength values (0.1, 0.5, 1, 2, and 3 M NaCl) were tested. The solutions’ pH was adjusted by the addition of 0.01 M HCl or 0.01 M NaOH. 

The apparent viscosity of the d-FucoPol aqueous solutions was determined at 25 °C (except for those meant for evaluating the effect of the temperature) using a controlled stress rheometer (Haake Mars III-Thermo Scientific, Karlsruhe, Germany), with a UTC-Peltier system to control the temperature, and a cone-plate sensor system (angle 2°, diameter 35 mm) and the shearing geometry covered with paraffin oil to prevent any water loss. The Cross model equation was used to fit the flow curves which were obtained [2]:

(1) $$\eta =\frac{\eta_{0}}{1+{(\tau \dot{\gamma })}^{m}}$$

where *η* is the apparent viscosity (Pa.s), *η*_0_ is the viscosity at a zero shear rate (Pa.s), *τ* (s) is the relaxation time, and *m* is a dimensionless constant, related to the exponent of power-law (*n*) by *m* = 1 − *n*. The frequency sweep tests were performed with a frequency ranging from 0.01 to 100 Hz, with a constant strain of 1% that was well within the linear viscoelastic limit, which was evaluated through preliminary amplitude sweep tests. 

### 2.5. Emulsion Forming and Stabilizing Capacity

The ability of d-FucoPol to stabilize the emulsions was assessed by mixing 3 mL of a polymer aqueous solution (1.0 wt%) with 2 mL of olive oil (purchased from a local market) to provide a 2:3 (*v*/*v*) emulsion ratio. The mixtures were manually agitated for 40 s and left standing for 24 h, at room temperature. The emulsification index after 24 h (*E*_24_, %) was determined using the following equation [4]:

(2) $$E_{24}=\frac{h_{e}}{h_{T}}\times 100$$

where h_e_ (mm) is the emulsion layer height and h_T_ (mm) is the total height of the mixture. The rheological properties of the emulsion were determined as described above.

### 2.6. Film-Forming Capacity

#### 2.6.1. Film Preparation

The d-FucoPol films were prepared following the procedure described by Freitas et al. [4]. Briefly, the d-FucoPol solutions (1.5 wt%) were prepared by dissolving the polymer in deionized water, under stirring for 12 h, at room temperature. Then, glycerol (86–88 wt%, Scharlau) was added as a plasticizer (30 wt%, on a dry basis). After removing the air bubbles under the vacuum, the solution was transferred to plastic Petri dishes (diameter of 6.5 cm) and dried at a temperature of 30 °C. The formed films were peeled from the dishes’ surface and conditioned at a controlled relative humidity (RH) of 58%, at 22 °C, for 48 h.

#### 2.6.2. Morphological Characterization

The films’ thickness was measured at three different points using a digital micrometre (Mitutoyo, Andover, UK). The films’ morphology was observed by Scanning Electron Microscopy (SEM) using an SEM Hitachi TM 3030Plus Tabletop (Krefeld, Germany). The dry film was fixed on the SEM stubs and coated with a thin layer of 15 nm Au/Pd. The observation of the samples was performed, in a secondary electron’s observation mode, at their surface and cross-section with an acceleration voltage of 15 kV. The films’ transparency was determined by measuring their transmittance at 600 nm (T600), using a UV-Vis spectrophotometer (CamSpec M509T, Leeds, UK), and calculated using the equation [5]:

(3) $$\mathrm{Transparency}\;=\frac{-{\;\mathrm{Log}\;T}_{600}}{\;X}$$

where *X* corresponds to the film’s thickness (mm).

#### 2.6.3. Mechanical Properties

The mechanical properties of the films were assessed under tensile tests at an ambient temperature (20 ± 1 °C), using a texture analyser TMS-Pro (Food Technology Corporation, Kent, UK) equipped with a 250 N load cell. The films were cut into rectangular-shaped strips (30 mm × 15 mm), attached to tensile grips A/TG, and stretched with a crosshead speed of 0.5 mm/s in tensile mode, until the breaking. The films’ stiffness was determined by the Young’s modulus (MPa), calculated as the slope of the linear initial section of the stress-strain curve. The tensile strength at the break was determined as the ratio of the maximum force to the films’ initial cross-sectional area. The elongation at the break (MPa) was determined as the ratio of the sample extension upon rupture by the initial gage length. Four replicas were analysed.

## 3. Results and Discussion

### 3.1. Optimization of the Chemical Deacylation Conditions

FucoPol deacetylation and desuccinylation studies in alkaline conditions were performed using several NaOH concentrations, temperatures, and reaction times (Table 1) to define the ones leading to the complete acetyl and succinyl groups’ removal. The original FucoPol sample had succinyl and acetyl contents of 1.1 wt% and 5.8 wt%, respectively, which are within the values reported in previous studies (0.6–3.0 wt% and 3.5–6.8 wt%, respectively). As shown in Table 1, acetyl was more easily removed from the polymer’s molecule as milder conditions achieved its complete removal. No acetate was detected in the samples treated with an alkali concentration of only 0.01 M, under 60 °C for 60 min, and at a lower temperature (40 °C) and for a shorter reaction time (15 min) for NaOH 0.02 M. As reported by Due et al. [26], the hydroxyl group performs a nucleophile attack in the electron-deficient carbonyl carbon of the acetyl group, resulting in the substitution of the acetyl group by a hydroxyl group. On the other hand, harsher conditions were required for the complete removal of succinyl, namely, a higher NaOH concentration (0.02 M) and a longer reaction time (≥30 min). Succinyl and pyruvyl have been reported to form strong intra-molecular hydrogen bonds, conferring a higher stability to their ester linkages compared to the acetyl groups [27], which might explain the faster removal of the acetyl groups from the FucoPol.

FucoPol depyruvylation was attempted by acid hydrolysis with oxalic acid and hydrochloride acid, following procedures described in the literature for pyruvate removal from xanthan gum [22,25]. However, all treatments in the acidic media were accompanied by a very low polymer recovery, which is probably inherent to the pyruvate branching position (2,3 linkage instead of 4,6 position, which is more often encountered) [3,22].

Given these results, treatment with NaOH 0.02 M at 60 °C for 15 min was selected as the most suitable to reach complete deacetylation and desuccinylation, while saving on alkali usage and the reaction time. The deacetylated and desuccinylated FucoPol was named d-FucoPol.

### 3.2. Physical and Chemical Characterization of the Deacetylated/Desuccinylated FucoPol

#### 3.2.1. Sugar Composition

The compositional analysis of d-FucoPol showed that the alkaline deacetylation/desuccinylation treatment had no significant impact on the polysaccharide’s monosaccharide structure, which remained identical to that of the original FucoPol sample. The fucose and galactose contents (37 and 27 mol%, respectively) remained essentially the same (36 and 28 mol%, respectively), while a slight increase in the glucose content was noticed for d-FucoPol (29 mol%, compared to 26 mol% for FucoPol). There was also a reduction in the content of glucuronic acid from 10 to 7 mol%. Deacetylation leads to the removal of acetyl protecting groups in glucuronic acid and to the formation of β-D-glucosiduronic acid, which could explain the slight decrease in the content of glucuronic acid detected after this chemical reaction [28]. The analysis showed a sugar content increase of approximately 8% from the polymer’s dry weight, which is in agreement with the removal of the substituents (acetate and succinate).

#### 3.2.2. Structural Analysis

**Figure 1:**
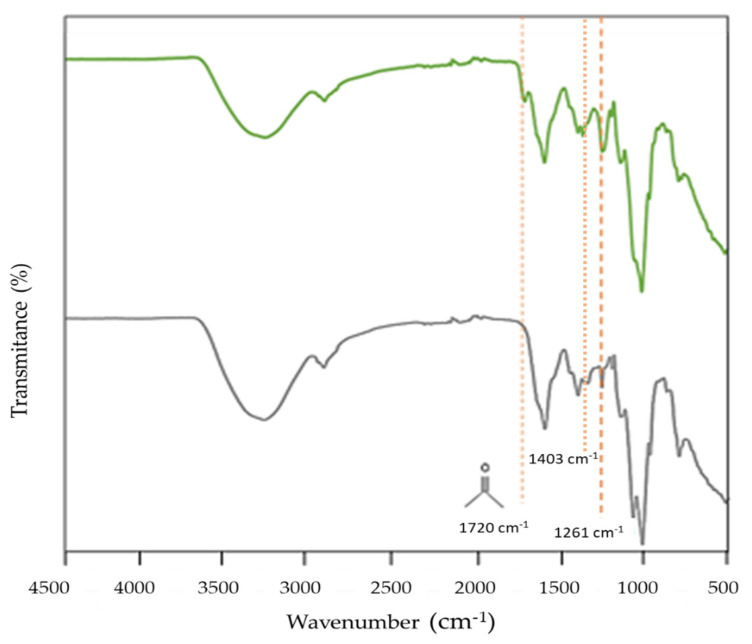
FT−IR spectra of FucoPol (green line) and d-FucoPol (grey line).

As shown in Figure 1, the FT-IR spectrum of d-FucoPol is similar to that reported in the literature for FucoPol [1,2,7], displaying peaks around 3283–2925 cm^−1^, which are attributed to the O-H stretching of hydroxyls’ vibrations and the C-H stretching peak of the CH_2_ groups; around 970–1145 cm^−1^, which are related to the C-C and C-O stretching in the pyranoid ring and C-O-C stretching of glycosidic bonds; and at 1603 cm^−1^, which is associated with the bending vibration of O-H, indicating bound water [2]. The main difference noticed for d-FucoPol is the absence of the peak at 1720 cm^−1^ (corresponding to the C=O stretching vibrations of carbonyls), and a noticeable peak intensity decrease at 1261 cm^−1^ (C-O-C vibrations), which are probably related to the removal of the acetyl e succinyl groups from the macromolecule [26,29,30,31]. Additionally, the peak appearing at 1403 cm^−1^, which may be assigned to the asymmetric and symmetric stretching of carboxylates from glucuronic acid [2], had a decreased intensity compared to the FucoPol spectrum, that might be correlated with the observed decrease in the polymer’s glucuronic content (from 10 to 7 mol%).

#### 3.2.3. Molecular Mass Distribution

d-FucoPol showed an average Mw of 2.7 × 10^6^ Da and a PDI of 1.7, which are within the ranges reported in previous studies (Mw = 1.7 × 10^6^ − 5.8 × 10^6^ Da; PDI = 1.3–1.9) [1,2,6,7,32]. Contrary results were obtained by Wang et al. [33] where the deacetylation reduced the molecular weight of native xanthan gum (from 1.1 × 10^6^ to 1.0 × 10^6^ Da) [33]. The same was reported for succinoglycan, where the removal process of the acetyl and succinyl groups decreased the molecular weight from 1.2 × 10^6^ to 1.1 × 10^6^ Da [17]. These findings confirm that the applied alkaline treatments had no significant impact on the biopolymer’s molecular mass distribution.

#### 3.2.4. Thermal Properties

**Figure 2:**
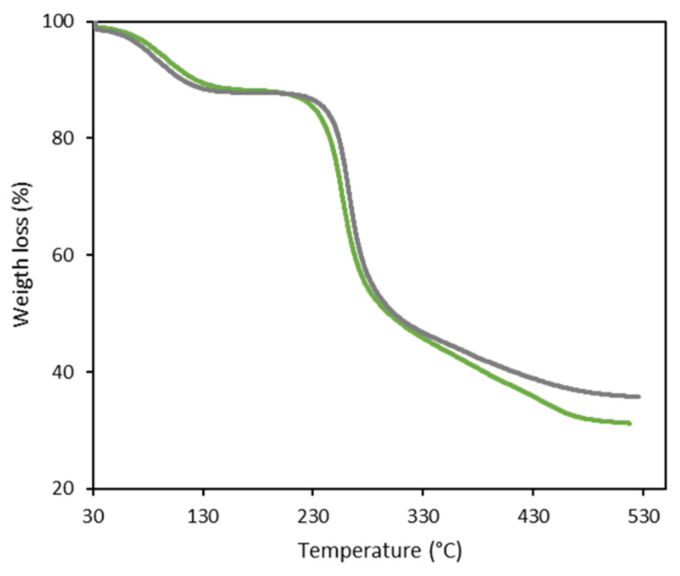
Thermogravimetric curves of FucoPol (green line) and d-FucoPol (grey line).

FucoPol and d-FucoPol displayed similar TGA curves, with two degradation steps (Figure 2) [2,6]. In the first degradation step, occurring between 40 and around 150–160 °C, similar weight losses were found for both samples (11–13%), which corresponded to the loss of the water adsorbed to the polysaccharides due to their hygroscopic nature [2]. The second and main degradation step occurred between 196 and 320 °C, with weight losses of around 41% for both samples, which can be attributed to the thermal depolymerization of the polysaccharides’ chains and the dehydration of the saccharide rings [34]. The T_deg_ value was similar for both FucoPol (259 °C) and d-FucoPol (256 °C), thus confirming no significant differences between them. For higher temperatures, there was a gradual reduction in the samples’ weight, resulting in char yields of 31–36% at 500 °C.

#### 3.2.5. Intrinsic Viscosity

The intrinsic viscosity, [*η*], of d-FucoPol was 2.45 ± 0.33 dL/g. This value is lower than that previously reported for FucoPol (11.0 dL/g) [32]. This fact can be correlated to a considerable difference in their chain stiffness. Deacylated FucoPol may have a weaker chain stiffness than FucoPol, decreasing the hydrodynamic volume occupied by the polymer chains, resulting in a lower [*η*] [35]. In addition, the removal of the succinyl groups leads to a lower number of negative charges on the polyelectrolyte d-FucoPol chains, decreasing the intra-chain charge repulsion, with a positive effect on the decrease in the chains’ hydrodynamic volume. A similar effect was reported by Wang et al. [33] for deacetylated xanthan that displayed an intrinsic viscosity of 43.3 dL/g, corresponding to a 42% decrease compared to native xanthan gum. A lower intrinsic viscosity can be an advantage if the intended application is conducive to absorption and diffusion in vivo, thus allowing an enhanced biological activity [36].

### 3.3. Apparent Viscosity and Viscoelastic Properties of the d-FucoPol Solutions

#### 3.3.1. Apparent Viscosity

**Figure 3:**
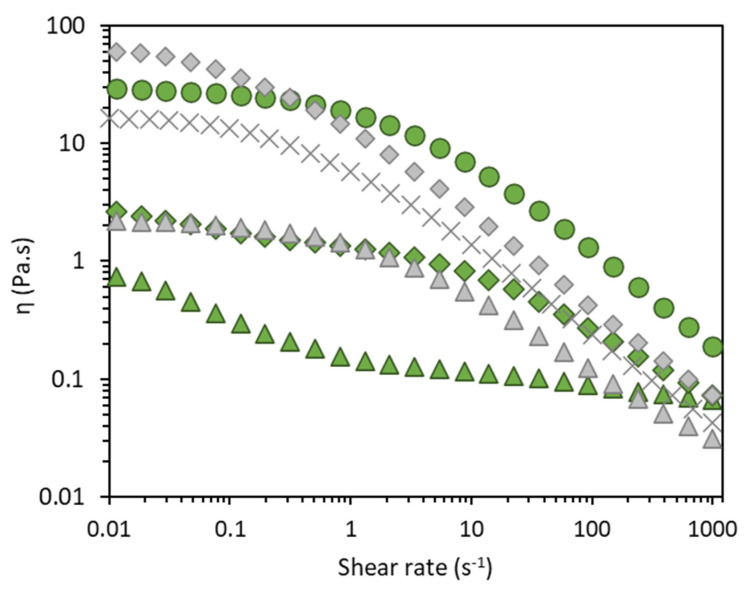
Flow curves of d−FucoPol (green) and FucoPol (grey) aqueous solutions: 0.75 wt% (triangles), 1.0 wt% (asterisk), 1.5 wt% (diamonds), and 3.0 wt% (circles).

d-FucoPol’s aqueous solutions displayed a shear-thinning behaviour (Figure 3), similar to that of FucoPol and typical of high molecular weight polysaccharides [2,37], for all three tested polymer concentrations (0.75, 1.5, and 3.0 wt%). A clear increase in the apparent viscosity is observed when increasing the polymer concentration which was tested. However, the thickening capacity of d-Fucopol was considerably lower than that of FucoPol (Figure 3). Indeed, the 1.5 wt% d-FucoPol solution displayed a zero-shear viscosity of 1.61 ± 0.01 Pa.s, which is considerably lower than the value (17.40 ± 0.04 Pa.s) found for a 1.0 wt% [2] and for 1.5 wt% (64.78 ± 0.31 Pa.s) FucoPol solution (Table 2). These results suggest that the acetyl and/or succinyl groups were probably responsible for promoting intermolecular interactions in the FucoPol solutions, contributing to its higher apparent viscosity [10], thus, their removal resulted in the observed lower viscosity. For both polymer types, higher *ƞ*_0_ values are associated with higher estimated *τ* values, representing higher relaxation times. The highest *η*_0_ value was observed for the 3.0 wt% d-FucoPol solution (29.5 ± 0.09 Pa.s) (Table 2), which was chosen for the subsequent tests for evaluating the effect of the ionic strength, pH, and temperature on d-FucoPol’s viscoelastic properties.

#### 3.3.2. Effect of Ionic Strength

**Figure 4:**
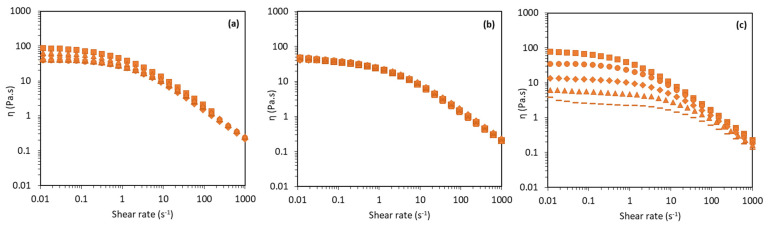
Flow curves of d-FucoPol aqueous solutions. (**a**) Effect of ionic strength: 0.1 M (dashed line), 0.5 M (circles), 1.0 M (diamonds), 2.0 M (triangles), and 3.0 M (squares). (**b**) Effect of pH: 3.6 (triangles), 4.8 (squares), 6.2 (circles), 8.5 (diamonds), and 11.5 (dashed line). (**c**) Effect of temperature: 10 °C (squares), 25 °C (circles), 45 °C (diamonds), 65 °C (triangles), and 80 °C (dashed line).

As shown in Figure 4a, although the d-FucoPol solutions kept a shear-thinning behaviour for all the ionic strength values which were tested (0.1, 0.5, 1.0, 2.0, and 3.0 M NaCl), increasing the NaCl concentration resulted in a higher *η_0_*, which increased from 26.1 ± 0.27 Pa.s for the 0.1 M solution to 86.9 ± 0.80 Pa.s for the 3.0 M solution, concomitant with a higher *τ* (from 0.62 ± 0.02 to 1.01 ± 0.04 s) and *m* (from 0.72 ± 0.01 to 0.76 ± 0.01). These results show that the d-FucoPol solutions’ viscosity was highly impacted by the ionic strength. This behaviour may result from the polymer aggregation probably induced by the higher salinity of the solution [38]. This behaviour of d-FucoPol contrasts with that of native FucoPol, whose viscosity was practically unaffected by the solution’s ionic strength within the range from 0.05 to 0.50 M NaCl [39].

**Figure 5:**
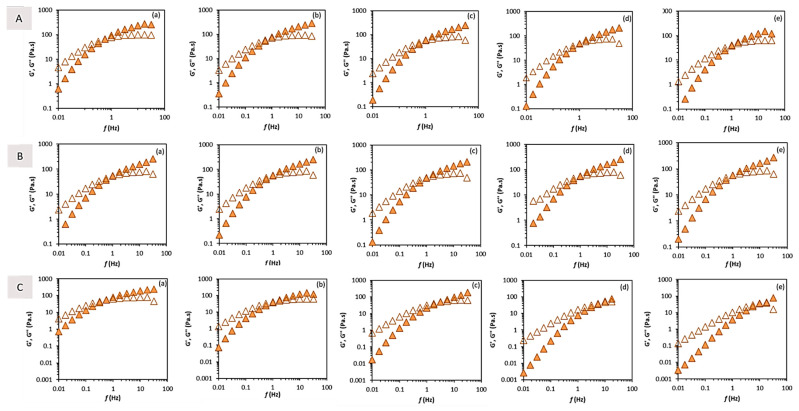
Mechanical spectra for d-FucoPol. (**A**) Ionic strength (NaCL) effect: (a) 3 M, (b) 2 M, (c) 1 M, (d) 0.1 M, and (e) 0.5 M. (**B**) pH: (a) 3.6, (b) 4.8, (c) 6.2, (d) 8.5, and (e) 11.5. (**C**) Temperature effect: (a) 10 °C, (b) 25 °C, (c) 45 °C, (d) 65 °C, and (e) 80 °C. Elastic G′ (closed) and viscous G″ (open) moduli.

For all the tested ionic strength values, at low frequencies, the loss modulus (G″) was higher than the storage modulus (G′) (Figure 5A), denoting a liquid-like behaviour for the biopolymer solution [2], with a crossover occurring at 1.0 Hz, except for the 3.0 M solution, in which a slight decrease was noticed (0.6 Hz) (Table 3). Both the G′ and G″ values increased for the higher ionic strength (Table 3), also with increasing gaps between the moduli. These results envisage stronger intermolecular interactions occurring for higher ionic strength values, being responsible for the reinforcement of the entangled polymer system [40].

#### 3.3.3. Effect of pH

In contrast with the FucoPol solutions, for which the apparent viscosity was reduced for high (pH ≥ 10.5) and low pH (pH ≤ 3) values [39], d-FucoPol solutions’ viscosity was not impacted by the solution’s pH within the tested range (3.6–11.5) (Figure 4b), with all solutions presenting similar *η*_0_ (40.1–44.7 Pa.s), *τ* (0.71–0.80 s), and *m* (0.73–0.78) values (Table 3). Possibly due to succinyl group elimination, the polymer chains became less susceptible to pH changes, presenting an interesting opposite behaviour to that of FucoPol whose solutions had decreased viscosity upon increasing the pH [39].

For all the pH values tested, at low frequencies, the loss modulus (G″) was higher than the storage modulus (G′) (Figure 5B), implying a liquid-like behaviour for the biopolymer solution [2], but the values of both the moduli and their crossover (1.0 Hz) remained unchanged for the entire range of the pH values tested (Table 3). The deacetylation apparently confers pH resistance to FucoPol aqueous solutions, which can be advantageous for some applications, such as, for example, drug delivery [41,42].

#### 3.3.4. Effect of Temperature

Similarly to the FucoPol solutions [32], increasing the temperature leads to the reduction in the d-FucoPol solutions’ apparent viscosity, despite maintaining their shear-thinning behaviour (Figure 4c). As shown in Table 3, the *η*_0_ decreased from 79.1 ± 0.46 to 2.66 ± 0.04 Pa.s, as the temperature increased from 10 to 80 °C. This effect was previously described by several authors, showing that high temperatures do not promote interactions between polymer molecules, which lowered the reported viscosity values [43,44,45]. In fact, the temperature increase enhances the molecular motion and reduces the intermolecular entanglements, thus resulting in a reduction in the solution’s apparent viscosity [44]. It is notorious in Figure 4c that the shear rate corresponding to the transition from Newtonian to shear-thinning behaviour moves to higher values as the temperature increases, which means that for higher temperatures, the formation of new interactions is faster. As expected, *η*_0_ and *τ* decreased with the increasing temperatures. The relaxation time (*τ*) decreased from 1.26 ± 0.02 to 0.06 ± 0.01 as the temperature increased from 10 to 85 °C. This fact indicates that less time is needed to form new interactions between polymer molecules at higher temperatures. Consequently, the transition from the Newtonian plateau to the shear-thinning regime is less evident and moves to the higher shear rate values [32].

As expected, the G″ > G′ at low frequencies, indicating a liquid-like behaviour (Table 3; Figure 5C). Moreover, the dynamic crossover, after which the elastic contribution is predominant, occurs at increasingly higher values as the temperature increases. At low temperatures (10 and 25 °C), the crossover occurred at lower frequencies (0.6–1.0 Hz), indicating a more entangled system storing most of the energy received [32,39]. However, at higher temperatures (65 and 80 °C), the crossover was perceived at 10 Hz (Table 3; Figure 5C(d,e)), showing a much lower number of polymer interactions needing a higher frequency energy input to notice a larger amount of energy stored compared to that dissipated [40].

### 3.4. Emulsion Forming and Stabilizing Capacity

**Figure 6:**
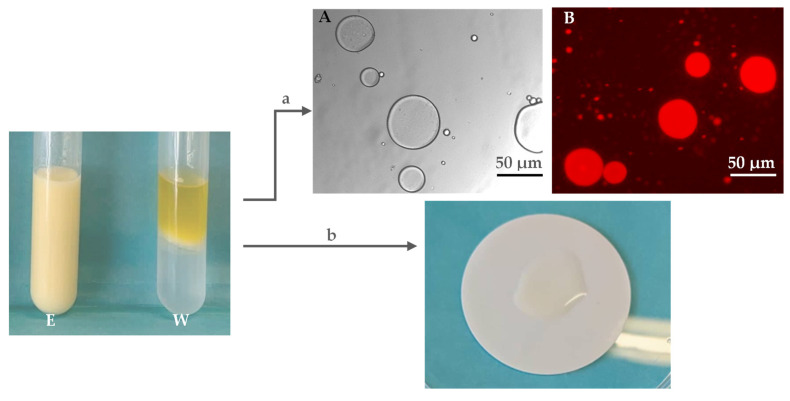
(a) Optical microscopic (40×) images of olive oil/d-FucoPol emulsion (E) (o:w weight ratio of 2:3); contrast phase and fluorescence after Nile Blue A staining (**A**,**B**, respectively). (b) Filter paper wetting test for emulsion type determination. (W)—blank sample, oil/water, and film obtained with deacylated form of FucoPol.

The ability of d-FucoPol to form and stabilize emulsions was assessed and compared with that of FucoPol, which has been reported as a good emulsifier for several oils [1,2,4]. The emulsions were prepared by mixing the d-FucoPol aqueous solution (1.0 wt%) with olive oil, the test hydrophobic compound, at an o:w weight ratio of 2:3. The results (Table 4) show that d-FucoPol had an identical emulsifying ability (E_24_ = 98%) to FucoPol (E_24_ = 81–98%) [2,46] under similar conditions. The microscopic observation (Figure 6a) of the emulsion displayed dispersed oil droplets in the aqueous phase, a characteristic of oil-in-water (O/W) emulsions [47,48]. Additionally, the emulsions’ droplets rapidly dispersed in the filter paper wetting test (Figure 6b), thus confirming its O/W nature [49,50,51].

**Figure 7:**
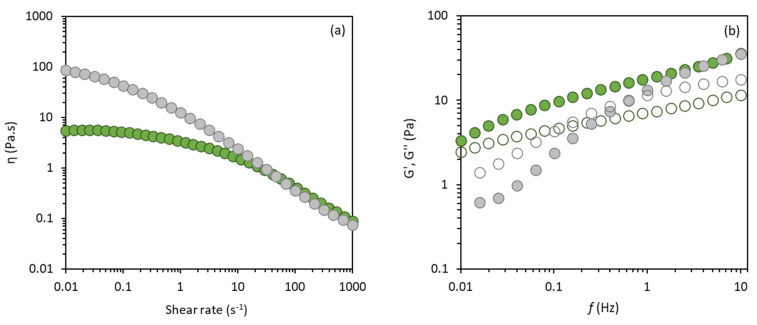
Rheological profile analysis of olive oil/d−FucoPol emulsion (green) and olive oil/FucoPol emulsion (grey) (o:w weight ratio of 2:3): (**a**) flow curves; (**b**) mechanical spectra: elastic G′ (closed) and viscous G″ (open) moduli.

As shown in Figure 7a, the emulsion stabilized by the d-FucoPol exhibited the shear-thinning flow behaviour characteristic of oil/FucoPol emulsions [2,39,46]. This flow behaviour is a common attribute of emulsions, in which the individual droplets and/or aggregated droplet clusters are deformed and disturbed by imposed forces at higher shear rates, leading to a reduction in both the flow resistance and viscosity [52]. The experimental results were fitted to the Cross model and the resulting parameters are given in Table 4. The *η*_0_ value observed for the d-FucoPol emulsion (13.92 ± 2.36 Pa.s) was lower than that of the FucoPol emulsion (46.5 ± 5.3 Pa.s) (Table 4), thus suggesting that the FucoPol’s acetyl and succinyl substituents are relevant for the emulsion to maintain a high apparent viscosity, as previously reported for chitin and for the polysaccharide from *Millettia speciosa* Champ [53,54]. In fact, the presence of acetyl groups is known to contribute to the reduction in interfacial tension and the increase in the rate of the molecules’ adsorption into the interface, which leads to the expeditious formation of smaller droplets [52]. This alteration in the viscosity of the interdroplet aqueous solution may be the main cause of variation in the emulsion’s viscosity [55].

Nevertheless, the d-FucoPol (1.0 wt%) stabilized emulsion still displayed a significantly higher viscosity (13.92 ± 2.36 Pa.s) than the 1.5 wt% polymer’s aqueous solution (1.61 ± 0.01 Pa.s) (Table 3). This fact is frequently caused by the formation of a network composed of polymer chains and oil droplets, leading to a higher resistance to the flow under a steady shear [56]. The same was observed for the emulsions stabilized with the native FucoPol [2].

Interestingly, the d-FucoPol stabilized emulsion presented the G′ higher than the G″ across the whole frequency range (0.01–10 Hz) (Figure 7b) and did not display any crossover, meaning that the oil/deacylated polymer emulsions displayed a gel-like behaviour [57,58]. Gel-like emulsions have been shown to be suitable for cosmetic applications because this elastic mechanical property favours the emulsion stability over long storage periods. Moreover, this structure was demonstrated to improve the moisturizing properties in emulsions with a shear-thinning behaviour [59]. This observation can be related to the formation of a three-dimensional network, supported by the development of an entangled network between absorbed and non-absorbed biopolymer molecules, which impacts the hydrocolloid properties of the emulsion [57,58,60]. This behaviour was not observed for the emulsions prepared with FucoPol in its native form [46] that presented a liquid-like behaviour, where the G″ > G′ for frequencies of 0.02–0.6 Hz (Figure 7b).

### 3.5. Film-Forming Capacity

#### 3.5.1. Morphological Characterization

**Figure 8:**
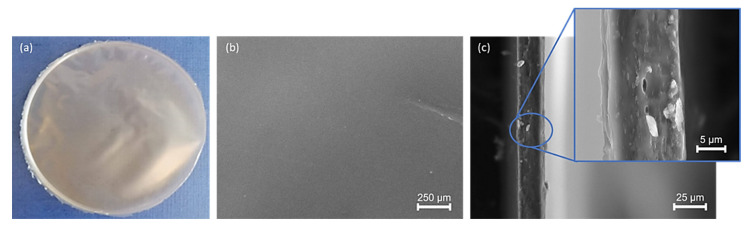
Film prepared with d−FucoPol (**a**) and its observation by SEM: surface (**b**) and cross-section (**c**) images.

The d-FucoPol formed slightly opaque flexible films with a brownish tone (Figure 8a) and a thickness of 45 ± 12 µm (Figure 8c). The films’ transparency was 5.16 ± 1.09, a value higher than that reported for the native FucoPol films (3.67) [5], thus indicating that the deacetylation and desuccinylation of the polysaccharide slightly increased its film opacity [61]. Transparency is important in packaging applications, providing see through properties and preventing light transmission [62,63], and in wound dressings to accurately monitor wound healing [64]. The films’ complete solubilization when placed in water indicated the inexistence of cross-linking reactions [5]. As shown by the SEM images, the films were compact (Figure 8c) with a smooth surface (Figure 8b). Regarding the FucoPol films’ morphology, the study of Ferreira et al. [65] revealed that it was constituted by a dense and irregular structure.

#### 3.5.2. Mechanical Properties

The d-FucoPol films showed a higher mechanical resistance (Table 5) compared to the films prepared with the native FucoPol, illustrated by the higher Young’s modulus (798 ± 152 MPa), concomitant with a higher stress at the break (22.46 ± 2.54 MPa) and a higher elongation at the break (9.27 ± 0.66%) [4]. According to Jin et al. [30], deacylation promotes the film’s strength and cohesiveness, which gives an indication of the film’s stiffness. In terms of the polysaccharide films’ mechanical properties, these are heavily determined by three factors: the intrinsic characteristics of the chosen biopolymer; the presence of additives (plasticizers, cross-linking agents, and film formation promoters); and the polymeric matrix water content which is present during the mechanical measurements [5,66]. For these reasons, the comparison of the films’ mechanical properties obtained in different investigations is challenging due to the subjective nature of the reported results which are usually obtained under specific conditions.

## 4. Conclusions

The acetyl and succinyl substituent groups were removed from FucoPol’s molecule by an alkaline treatment under the mild conditions of the temperature and alkali concentration. Although the deacetylated/desuccinylated polysaccharide, d-FucoPol, maintained the same sugar composition and molecular mass distribution as the native FucoPol, there was a significant reduction in the d-FucoPol aqueous solutions’ apparent viscosity, which was also significantly affected by the ionic strength of the solution and the temperature. Nevertheless, the solutions’ viscosity was not impacted by the pH, contrarily to the native FucoPol solutions. d-FucoPol kept the ability to form and stabilize the emulsions, although the resulting emulsions had a lower apparent viscosity. Additionally, the film-forming capacity of the FucoPol was also kept, presenting a higher mechanical resistance than the native FucoPol films. Given these features, d-FucoPol presents itself as a promising FucoPol derivative with distinctive properties that render it valuable for several applications, including in packaging, cosmetics, and in other areas as a suspending, thickening, or emulsifying agent.

## Figures and Tables

**Table 1:** Acyl groups (succinyl, acetyl, and pyruvyl) contents of FucoPol sample subjected to alkaline treatment.

Acyls Groups (wt%)	NaOH (M)	40 °C			60 °C		
		15 (min)	30 (min)	60 (min)	15 (min)	30 (min)	60 (min)
Succinyl	0.005	0.70 ± 0.02	0.67 ± 0.02	0.62 ± 0.01	0.65 ± 0.08	0.64 ± 0.02	0.47 ± 0.01
	0.01	0.35 ± 0.02	0.26 ± 0.04	0.27 ± 0.04	0.34 ± 0.02	0.29 ± 0.00	0.22 ± 0.04
	0.02	0.19 ± 0.00	-	-	-	-	-
	0.05	-	-	-	-	-	-
Acetyl	0.005	3.15 ± 0.22	3.02 ± 0.07	2.55 ± 0.10	2.90 ± 0.38	2.87 ± 0.08	1.85 ± 0.01
	0.01	1.41 ± 0.15	1.16 ± 0.06	0.61 ± 0.10	1.05 ± 0.17	0.44 ± 0.05	-
	0.02	-	-	-	-	-	-
	0.05	-	-	-	-	-	-
Pyruvyl	0.005	3.61 ± 0.35	3.58 ± 0.04	3.580 ± 0.5	3.96 ± 0.27	4.05 ± 0.29	4.01 ± 0.27
	0.01	3.68 ± 0.30	3.52 ± 0.11	3.77 ± 0.53	3.63 ± 0.47	3.68 ± 0.29	4.03 ± 0.62
	0.02	3.63 ± 0.05	3.30 ± 0.00	3.50 ± 0.22	3.54 ± 0.21	4.00 ± 0.12	3.99 ± 0.55
	0.05	3.65 ± 0.64	3.45 ± 0.50	3.90 ± 0.14	3.73 ± 0.09	3.89 ± 0.24	3.88 ± 0.45

**Table 2:** Cross model parameters estimated for d-FucoPol and Fucopol solutions. *η*_0_—apparent viscosity of the first Newtonian plateau (Pa·s); *τ*—relaxation time (s); and *m*—dimensionless constant. Data are shown as the average ± standard deviation (SD) (n = 3). * Cross model is not appropriate to describe experimental data.

Sample	Concentration (wt%)	Cross Model Parameters			Reference
		*η*_0_ (Pa.s)	*τ* (s)	*m*	
d-FucoPol	0.75 1.5 3.0	- * 1.61 ± 0.01 29.5 ± 0.09	- * 0.11 ± 0.00 0.55 ± 0.01	- * 0.67 ± 0.01 0.75 ± 0.01	This study
FucoPol	0.75 1.0 1.5	2.20 ± 0.01 17.40 ± 0.04 64.78 ± 0.31	0.51 ± 0.00 1.68 ± 0.21 5.66 ± 0.15	0.72 ± 0.00 0.78 ± 0.00 0.83 ± 0.02	This study [2] This study

**Table 3:** Cross model parameters estimated for d-FucoPol aqueous solutions (3.0 wt%) tested at different ionic strength, pH, and temperatures. *η*_0_—apparent viscosity of the first Newtonian plateau; *τ*—relaxation time; and *m*—dimensionless constant. Data are shown as the average ± standard deviation (SD) (n = 3). Viscoelastic parameters estimated for d-FucoPol (3.0 wt%). G′—storage/elastic modulus and G″—loss/viscous modulus at *f* = 0.1 Hz.

Parameter	Cross Model			Viscoelastic Parameters		
	*η*_0_ (Pa.s)	*τ* (s)	*m*	G′ (Pa)	G″ (Pa)	Crossover (Hz)
Ionic strength (M) (pH = 6.2, 25 °C)						
0.1 0.5 1.0 2.0 3.0	36.2 ± 0.12 40.1 ± 0.16 47.9 ± 0.93 65.6 ± 0.56 86.9 ± 0.80	0.54 ± 0.01 0.51 ± 0.01 0.91 ± 0.06 0.92 ± 0.03 1.01 ± 0.04	0.74 ± 0.00 0.78 ± 0.01 0.73 ± 0.01 0.75 ± 0.01 0.76 ± 0.01	4.05 5.40 7.40 10.9 15.5	11.3 14.3 18.1 23.7 29.5	1.0 1.0 1.0 1.0 0.6
pH (0.1 M, 25 °C)						
3.6 4.8 6.2 8.5 11.5	40.4 ± 0.44 44.7 ± 0.47 40.1 ± 0.16 41.0 ± 0.45 40.6 ± 0.33	0.71 ± 0.03 0.80 ± 0.03 0.71 ± 0.01 0.75 ± 0.03 0.74 ± 0.02	0.74 ± 0.01 0.73 ± 0.01 0.78 ± 0.00 0.75 ± 0.03 0.74 ± 0.01	7.15 7.65 5.40 6.98 6.70	16.6 17.8 14.3 17.4 17.1	1.0 1.0 1.0 1.0 1.0
Temperature (°C) (0.1 M, pH = 6.2)						
10 25 45 65 80	79.1 ± 0.46 36.2 ± 0.12 13.0 ± 0.05 5.83 ± 0.05 2.66 ± 0.04	1.26 ± 0.02 0.54 ± 0.01 0.21 ± 0.06 0.11 ± 0.01 0.06 ± 0.01	0.76 ± 0.01 0.77 ± 0.00 0.76 ± 0.01 0.69 ± 0.02 0.67 ± 0.04	13.5 4.05 1.31 0.22 0.12	25.1 11.3 6.31 2.42 1.39	0.6 1.0 3.2 10 10

**Table 4:** Emulsification index (E_24_) and Cross model parameters estimated for the emulsions prepared with the deacylated FucoPol and olive oil, at an oil/water (O/W) weight ratio of 3:2: *η*_0_—apparent viscosity of the first Newtonian plateau (Pa·s); *τ*—relaxation time (s); and *m*—dimensionless constant.

Sample	E_24_ (%)	Cross Model Parameters			Reference
		*η*_0_ (Pa.s)	*τ* (s)	*m*	
d-FucoPol	98 ± 0	13.9 ± 2.4	1.64 ± 0.13	0.74 ± 0.00	This study
FucoPol	81–98	46.5 ± 5.3	-	-	[2,46]

RE = $\overset{n}{\underset{i=1}{\sum }}\left(\left|\mathrm{xeI}–,i\;-\;\mathrm{xcalc},i\right|/\mathrm{xexp}\right)/n\;\;$ is between 0.011 and 0.019.

**Table 5:** Mechanical parameters and transparency of the films prepared with d-FucoPol, FucoPol, and other biopolymers. Data are shown as the average ± standard deviation (SD) (n = 4).

Sample	Mechanical Parameters			Transparency	References
	Young Modulus (MPa)	Stress at Break (MPa)	Elongation at Break (%)		
d-FucoPol	798 ± 152	22.5 ± 2.5	9.3 ± 0.7	5.16 ± 1.09	This study
FucoPol	458 ± 32	15.5 ± 0.3	8.1 ± 1.0	3.67 ± 0.57	[4,5]
GalactoPol	1738 ± 114	51.0 ± 3.0	9.5 ± 3.9	-	[67]
Chitosan	21.8 ± 4.06	8.90 ± 1.6	38.5 ± 5.2	-	[68]

## Data Availability

Data will be available upon request.

## Sample Availability

Samples of the compounds d−FucoPol and FucoPol are available from the authors.

## References

[1] Freitas F., Alves V.D., Torres C.A.V., Cruz M., Sousa I., João M., Ramos A.M., Reis M.A.M. (2011). Fucose-Containing Exopolysaccharide Produced by the Newly Isolated *Enterobacter* strain A47 DSM 23139. Carbohydr. Polym..

[2] Baptista S., Torres C.A.V., Sevrin C., Grandfils C., Reis M.A.M., Freitas F. (2022). Extraction of the Bacterial Extracellular Polysaccharide FucoPol by Membrane-Based Methods: Efficiency and Impact on Biopolymer Properties. Polymers.

[3] Guerreiro B.M., Freitas F., Lima J.C., Silva J.C., Dionísio M., Reis M.A.M. (2020). Demonstration of the Cryoprotective Properties of the Fucose-Containing Polysaccharide FucoPol. Carbohydr. Polym..

[4] Freitas F., Alves V.D., Gouveia A.R., Pinheiro P., Torres C.A.V., Grandfils C., Reis M.A.M. (2014). Controlled Production of Exopolysaccharides from *Enterobacter* A47 as a Function of Carbon Source with Demonstration of Their Film and Emulsifying Abilities. Appl. Biochem. Biotechnol..

[5] Ferreira A.R.V., Torres C.A.V., Freitas F., Reis M.A.M., Alves V.D., Coelhoso I.M. (2014). Biodegradable Films Produced from the Bacterial Polysaccharide FucoPol. Int. J. Biol. Macromol..

[6] Fialho L., Araújo D., Alves V.D., Roma-Rodrigues C., Baptista V., Fernandes A.R., Freitas F., Reis M.A.M. (2021). Cation-Mediated Gelation of the Fucose-Rich Polysaccharide FucoPol: Preparation and Characterization of Hydrogel Beads and Their Cytotoxicity Assessment. Int. J. Polym. Mater. Polym. Biomater..

[7] Concórdio-Reis P., Pereira C.V., Batista M.P., Sevrin C., Grandfils C., Marques A.C., Fortunato E., Gaspar F.B., Matias A.A., Freitas F. (2020). Silver Nanocomposites Based on the Bacterial Fucose-Rich Polysaccharide Secreted by *Enterobacter* A47 for Wound Dressing Applications: Synthesis, Characterization and in Vitro Bioactivity. Int. J. Biol. Macromol..

[8] Guerreiro B.M., Freitas F., Lima J.C., Silva J.C., Reis M.A.M. (2021). Photoprotective Effect of the Fucose-Containing Polysaccharide FucoPol. Carbohydr. Polym..

[9] Guerreiro B.M., Silva J.C., Lima J.C., Reis M.A.M., Freitas F. (2021). Antioxidant Potential of the Bio-Based Fucose-Rich Polysaccharide FucoPol Supports Its Use in Oxidative Stress-Inducing Systems. Polymers.

[10] Xu L., Qiu Z., Gong H., Zhu C., Li Z., Li Y., Dong M. (2019). Rheological Behaviors of Microbial Polysaccharides with Different Substituents in Aqueous Solutions: Effects of Concentration, Temperature, Inorganic Salt and Surfactant. Carbohydr. Polym..

[11] Jeong J.P., Kim Y., Hu Y., Jung S. (2022). Bacterial Succinoglycans: Structure, Physical Properties, and Applications. Polymers.

[12] Bajaj I.B., Survase S.A., Saudagar P.S., Singhal R.S. (2007). Gellan Gum: Fermentative Production, Downstream Processing and Applications. Food Technol. Biotechnol..

[13] Huang G., Xie J., Shuai S., Wei S., Chen Y., Guan Z., Zheng Q., Yue P., Wang C. (2021). Nose-to-Brain Delivery of Drug Nanocrystals by Using Ca^2+^ Responsive Deacetylated Gellan Gum Based in Situ-Nanogel. Int. J. Pharm..

[14] Wang L., Xiang D., Li C., Zhang W., Bai X. (2022). Effects of Deacetylation on Properties and Conformation of Xanthan Gum. J. Mol. Liq..

[15] Riaz T., Iqbal M.W., Jiang B., Chen J. (2021). A Review of the Enzymatic, Physical, and Chemical Modification Techniques of Xanthan Gum. Int. J. Biol. Macromol..

[16] Veiga-Santos P., Oliveira L.M., Cereda M.P., Alves A.J., Scamparini A.R.P. (2005). Mechanical Properties, Hydrophilicity and Water Activity of Starch-Gum Films: Effect of Additives and Deacetylated Xanthan Gum. Food Hydrocoll..

[17] Ridout M.J., Brownsey G.J., York G.M., Walker G.C., Morris V.J. (1997). Effect of O-Acyl Substituents on the Functional Behaviour of *Rhizobium meliloti* Succinoglycan. Int. J. Biol. Macromol..

[18] Abdou E.S., Nagy K.S.A., Elsabee M.Z. (2008). Extraction and Characterization of Chitin and Chitosan from Local Sources. Bioresour. Technol..

[19] Knidri H., Belaabed R., Addaou A., Laajeb A., Lahsini A. (2018). Extraction, Chemical Modification and Characterization of Chitin and Chitosan. Int. J. Biol. Macromol..

[20] Kjartansson G.T., Zivanovic S., Kristbergsson K., Weiss J. (2006). Sonication-Assisted Extraction of Chitin from North Atlantic Shrimps (*Pandalus borealis*). J. Agric. Food Chem..

[21] Guetta O., Mazeau K., Auzely R., Milas M., Rinaudo M. (2003). Structure and Properties of a Bacterial Polysaccharide Named Fucogel. Biomacromolecules.

[22] Pinto E.P., Furlan L., Vendruscolo C.T. (2011). Chemical Deacetylation Natural Xanthan (Jungbunzlauer^®^). Polímeros.

[23] Torres C.A.V., Marques R., Antunes S., Alves V.D., Sousa I., Maria A., Oliveira R., Freitas F., Reis M.A.M. (2011). Kinetics of Production and Characterization of the Fucose-Containing Exopolysaccharide from *Enterobacter* A47. J. Biotechnol..

[24] Lima C.S., Rabelo S.C., Ciesielski P.N., Roberto I.C., Rocha G.J.M., Driemeier C. (2018). Multiscale Alterations in Sugar Cane Bagasse and Straw Submitted to Alkaline Deacetylation. ACS Sustain. Chem. Eng..

[25] Herasimenka Y., Cescutti P., Impallomeni G., Rizzo R. (2007). Exopolysaccharides Produced by *Inquilinus limosus*, a New Pathogen of Cystic Fibrosis Patients: Novel Structures with Usual Components. Carbohydr. Res..

[26] Du X., Li J., Chen J., Li B. (2012). Effect of Degree of Deacetylation on Physicochemical and Gelation Properties of Konjac Glucomannan. Food Res. Int..

[27] Kwon C., Lee S., Jung S. (2011). Matrix-Assisted Laser Desorption/Ionization Time-of-Flight Mass Spectrometric Behavior of Succinoglycan Monomers, Dimers, and Trimers Isolated from *Sinorhizobium meliloti* 1021. Carbohydr. Res..

[28] Marsh C.A., Dutton G.J. (1966). Chemistry of D-Glucuronic Acid and Its Glycosides. Glucuronic Acid Free and Combined.

[29] Chokboribal J., Tachaboonyakiat W., Sangvanich P., Ruangpornvisuti V., Jettanacheawchankit S., Thunyakitpisal P. (2015). Deacetylation Affects the Physical Properties and Bioactivity of Acemannan, an Extracted Polysaccharide from *Aloe vera*. Carbohydr. Polym..

[30] Jin W., Song R., Xu W., Wang Y., Li J., Shah B.R., Li Y., Li B. (2015). Analysis of Deacetylated Konjac Glucomannan and Xanthan Gum Phase Separation by Film Forming. Food Hydrocoll..

[31] Du X., Zhang J., Lv Z., Ye L., Yang Y., Tang Q. (2014). Chemical Modification of an Acidic Polysaccharide (TAPA1) from *Tremella aurantialba* and Potential Biological Activities. Food Chem..

[32] Cruz M., Freitas F., Torres C.A.V., Reis M.A.M., Alves V.D. (2011). Influence of Temperature on the Rheological Behavior of a New Fucose-Containing Bacterial Exopolysaccharide. Int. J. Biol. Macromol..

[33] Wang F., Wang Y.J., Sun Z. (2002). Conformational Role of Xanthan Gum in Its Interaction with Guar Gum. J. Food Sci..

[34] Wang J., Salem D.R., Sani R.K. (2021). Two New Exopolysaccharides from a Thermophilic Bacterium *Geobacillus* sp. WSUCF1: Characterization and Bioactivities. New Biotechnol..

[35] Khouryieh H.A., Herald T.J., Aramouni F., Alavi S. (2007). Intrinsic Viscosity and Viscoelastic Properties of Xanthan/Guar Mixtures in Dilute Solutions: Effect of Salt Concentration on the Polymer Interactions. Food Res. Int..

[36] Li S., Xiong Q., Lai X., Li X., Wan M., Zhang J., Yan Y., Cao M., Lu L., Guan J. (2016). Molecular Modification of Polysaccharides and Resulting Bioactivities. Compr. Rev. Food Sci. Food Saf..

[37] Ayyash M., Stathopoulos C., Abu-Jdayil B., Esposito G., Baig M., Turner M.S., Baba A.S., Apostolopoulos V., Al-Nabulsi A., Osaili T. (2020). Exopolysaccharide Produced by Potential Probiotic *Enterococcus faecium* MS79: Characterization, Bioactivities and Rheological Properties Influenced by Salt and pH. LWT.

[38] Madruga L.Y.C., Da Câmara P.C.F., Marques N.D.N., Balaban R.D.C. (2018). Effect of Ionic Strength on Solution and Drilling Fluid Properties of Ionic Polysaccharides: A Comparative Study between Na-Carboxymethylcellulose and Na-Kappa-Carrageenan Responses. J. Mol. Liq..

[39] Torres C.A.V., Ferreira A.R.V., Freitas F., Reis M.A.M., Coelhoso I., Sousa I., Alves V.D. (2015). Rheological Studies of the Fucose-Rich Exopolysaccharide FucoPol. Int. J. Biol. Macromol..

[40] Zhou F., Wu Z., Chen C., Han J., Ai L., Guo B. (2014). Exopolysaccharides Produced by *Rhizobium radiobacter* S10 in Whey and Their Rheological Properties. Food Hydrocoll..

[41] Barbosa A.I., Coutinho A.J., Costa Lima S.A., Reis S. (2019). Marine Polysaccharides in Pharmaceutical Applications: Fucoidan and Chitosan as Key Players in the Drug Delivery Match Field. Mar. Drugs.

[42] Singhvi G., Hans N., Shiva N., Kumar Dubey S., Hasnain M.S., Nayak A.K. (2019). Xanthan Gum in Drug Delivery Applications. Natural Polysaccharides in Drug Delivery and Biomedical Applications.

[43] Li L., Liao B.Y., Thakur K., Zhang J.G., Wei Z.J. (2018). The Rheological Behavior of Polysaccharides Sequential Extracted from *Polygonatum cyrtonema* Hua. Int. J. Biol. Macromol..

[44] Ji Y.H., Liao A.M., Huang J.H., Thakur K., Li X.L., Hu F., Wei Z.J. (2019). The Rheological Properties and Emulsifying Behavior of Polysaccharides Sequentially Extracted from *Amana edulis*. Int. J. Biol. Macromol..

[45] Tako M., Nakamura S. (1984). Rheological Properties of Deacetylated Xanthan. Agric. Biol. Chem..

[46] Baptista S., Pereira J.R., Gil C.V., Torres C.A.V., Reis M.A.M., Freitas F. (2022). Development of Olive Oil and α-Tocopherol Containing Emulsions Stabilized by FucoPol: Rheological and Textural Analyses. Polymers.

[47] Mcclements D.J. (2007). Critical Review of Techniques and Methodologies for Characterization of Emulsion Stability. Crit. Rev. Food Sci. Nutr..

[48] Akbari S., Nour A.H. (2018). Emulsion Types, Stability Mechanisms and Rheology: A Review. Int. J. Innov. Res. Sci. Stud..

[49] Kavitake D., Balyan S., Devi P.B., Shetty P.H. (2020). Evaluation of Oil-in-Water (O/W) Emulsifying Properties of Galactan Exopolysaccharide from *Weissella confusa* KR780676. J. Food Sci. Technol..

[50] Lata Yadav K., Kumar Rahi D., Kumar Soni S. (2014). Bioemulsifying Potential of Exopolysaccharide Produced by an Indigenous Species of *Aureobasidium pullulans* RYLF10. PeerJ.

[51] Kavitake D., Balyan S., Devi P.B., Shetty P.H. (2019). Interface between Food Grade Flavour and Water Soluble Galactan Biopolymer to Form a Stable Water-in-Oil-in-Water Emulsion. Int. J. Biol. Macromol..

[52] Huang Z., Zong M.H., Lou W.Y. (2022). Effect of Acetylation Modification on the Emulsifying and Antioxidant Properties of Polysaccharide from *Millettia speciosa* Champ. Food Hydrocoll..

[53] Zhang F., Cai X., Ding L., Wang S. (2021). Effect of pH, Ionic Strength, Chitosan Deacetylation on the Stability and Rheological Properties of O/W Emulsions Formulated with Chitosan/Casein Complexes. Food Hydrocoll..

[54] Li X., Xia W. (2011). Effects of Concentration, Degree of Deacetylation and Molecular Weight on Emulsifying Properties of Chitosan. Int. J. Biol. Macromol..

[55] Blanco L.F., Rodriguez M.S., Schulz P.C., Agulló E. (1999). Influence of the Deacetylation Degree on Chitosan Emulsification Properties. Colloid Polym. Sci..

[56] Calero N., Muñoz J., Cox P.W., Heuer A., Guerrero A. (2013). Influence of Chitosan Concentration on the Stability, Microstructure and Rheological Properties of O/W Emulsions Formulated with High-Oleic Sunflower Oil and Potato Protein. Food Hydrocoll..

[57] Bom S., Fitas M., Martins A.M., Pinto P., Ribeiro H.M., Marto J. (2020). Replacing Synthetic Ingredients by Sustainable Natural Alternatives: A Case Study Using Topical O/W Emulsions. Molecules.

[58] Heydari A., Razavi S.M.A. (2021). Evaluating High Pressure-Treated Corn and Waxy Corn Starches as Novel Fat Replacers in Model Low-Fat O/W Emulsions: A Physical and Rheological Study. Int. J. Biol. Macromol..

[59] Zhang W., Liu L. (2013). Study on the Formation and Properties of Liquid Crystal Emulsion in Cosmetic. J. Cosmet. Dermatol. Sci. Appl..

[60] Gamonpilas C., Pongjaruvat W., Fuongfuchat A., Methacanon P., Seetapan N., Thamjedsada N. (2011). Physicochemical and Rheological Characteristics of Commercial Chili Sauces as Thickened by Modified Starch or Modified Starch/Xanthan Mixture. J. Food Eng..

[61] Hoque M.S., Benjakul S., Prodpran T. (2010). Effect of Heat Treatment of Film-Forming Solution on the Properties of Film from Cuttlefish (*Sepia pharaonis*) Skin Gelatin. J. Food Eng..

[62] Khairunnisa S., Junianto J., Zahidah Z., Rostini I. (2018). The Effect of Glycerol Concentration as a Plasticizer on Edible Films Made from Alginate towards Its Physical Characteristic. World Sci. News.

[63] Khoirunnisa A.R., Joni I.M., Panatarani C., Rochima E., Praseptiangga D. (2018). UV-Screening, Transparency and Water Barrier Properties of Semi Refined Iota Carrageenan Packaging Film Incorporated with ZnO Nanoparticles. AIP Conf. Proc..

[64] Kędzierska M., Blilid S., Miłowska K., Kołodziejczyk-Czepas J., Katir N., Lahcini M., El Kadib A., Bryszewska M. (2021). Insight into Factors Influencing Wound Healing Using Phosphorylated Cellulose-Filled-Chitosan Nanocomposite Films. Int. J. Mol. Sci..

[65] Ferreira A.R.V., Torres C.A.V., Freitas F., Sevrin C., Grandfils C., Reis M.A.M., Alves V.D., Coelhoso I.M. (2016). Development and Characterization of Bilayer Films of FucoPol and Chitosan. Carbohydr. Polym..

[66] Kurt A. (2019). Rheology of Film-Forming Solutions and Physical Properties of Differently Deacetylated Salep Glucomannan Film. Food Health.

[67] Alves V.D., Ferreira A.R., Costa N., Freitas F., Reis M.A.M., Coelhoso I.M. (2011). Characterization of Biodegradable Films from the Extracellular Polysaccharide Produced by *Pseudomonas oleovorans* Grown on Glycerol Byproduct. Carbohydr. Polym..

[68] Kurek M., Galus S., Debeaufort F. (2014). Surface, Mechanical and Barrier Properties of Bio-Based Composite Films Based on Chitosan and Whey Protein. Food Packag. Shelf Life.

